# Commercial symptom monitoring devices in Parkinson’s disease: benefits, limitations, and trends

**DOI:** 10.3389/fneur.2024.1470928

**Published:** 2024-12-23

**Authors:** Daniel Rodríguez-Martín, Carlos Pérez-López

**Affiliations:** ^1^Sense4Care, Cornellà de Llobregat, Spain; ^2^Consorci Sanitari Alt Penedès-Garraf, Vilanova i la Geltrú, Spain

**Keywords:** wearables, motor symptoms, early detection, medical devices, objective evaluation

## Abstract

Parkinson’s disease (PD) is a neurodegenerative disorder that significantly impacts patients’ quality of life. Managing PD requires accurate assessment of motor and non-motor symptoms, often complicated by the subjectivity in symptom reporting and the limited availability of neurologists. To address these challenges, commercial wearable devices have emerged to continuously monitor PD symptoms outside the clinical setting. The main devices include PKG™, Kinesia 360™, Kinesia U™, PDMonitor™, and STAT-ON™. These devices utilize advanced technologies such as accelerometers, gyroscopes, and specific algorithms to provide objective data on motor symptoms like tremors, dyskinesia, and bradykinesia. Despite their potential, the adoption of these devices is limited due to concerns about their accuracy, complexity of use, and lack of independent validation. The correlation between these devices’ measurements and traditional clinical observations varies, and patient usability and adherence remain critical areas for improvement. To optimize their utility and improve patient outcomes, it is essential to conduct validation and usability studies with a sufficient number of patients, develop standardized protocols, and ensure integration with hospital information systems.

## 1 Introduction

Parkinson’s disease (PD) currently poses a global health challenge affecting millions of people, with its prevalence steadily increasing over the past decades (1–5). This neurodegenerative disorder is characterized by a wide range of symptoms, both motor and non-motor. Motor symptoms, including tremors, rigidity, and bradykinesia, profoundly impact patients’ daily lives (6). Despite significant advances in research, the management of Parkinson’s remains complex and highly dependent on the neurologist’s expertise and the accuracy of the patient’s symptom reporting. This dependency is exacerbated by the shortage of neurologists in many regions worldwide, especially in rural areas where access to movement disorder specialists is limited. Consequently, many patients must travel long distances regularly to receive specialized care, imposing a significant additional burden. This situation results in a considerable number of PD patients being managed by primary care physicians rather than neurologists. In developed countries such as Germany and the United States, over 40% of Parkinson’s patients are treated by primary care physicians (7).

Setting aside the professional’s experience in treating the patient, all healthcare providers rely on the patient’s subjective account and medical history during consultations, introducing bias in the information provided. Due to the nature of the disease and its symptoms, patients struggle to identify and distinguish specific symptoms, often confusing them (8–10). Furthermore, the patient’s account is heavily influenced by their mood at the time of the consultation, tending to report positive periods when they feel well and vice versa. This bias is often mitigated with comments or opinions from caregivers. However, both the patient and the caregiver tend to overlook mild symptoms or those with little impact on daily life, especially when recalling extended periods. Although these symptoms may seem less relevant in routine clinical practice, they are of great importance in clinical trials of new therapies or in evaluating new approaches to symptoms. The accurate assessment of mild and fluctuating symptoms, as well as their evolution over time, is of great scientific interest (7).

In response to these challenges, wearable devices emerge as highly promising tools in the management of PD. Specifically designed to assess PD, these devices offer a new approach by providing quantitative data on motor symptoms outside traditional clinical settings (11–13). These systems can continuously measure symptoms over extended periods in ambulatory conditions, providing neurologists with objective information on disease progression and treatment responses, enabling more informed decision-making and personalized interventions.

Despite their potential, the adoption of these devices remains limited. The main barrier to adoption lies in professionals’ low confidence in these solutions, which, according to the authors’ experience, is due to two clear factors. Firstly, many of the tools published or launched on the market have questionable validation studies and seem to have serious issues with false positives for the symptoms they attempt to measure. This factor, combined with the increasing number of available tools and the ensuing “commercial war” that floods professionals with information, leads to rejection and distrust towards these types of tools. Secondly, there is a tendency to maintain the classical measurement variables to assess the patient’s evolution. A clear example is the patient’s OFF time, a measure that presents multiple biases due to its definition and the way it is collected. Professionals tend to give more credibility to the patient’s account than to objective measures, including patient diaries. In a recent study, it was shown how low the correlation was between clinician observation and PD diaries (14, 15). This means that a neurologist may not adjust a patient’s treatment if the patient claims to be doing well, even if objective measures or diaries show that the patient experiences motor impairments for much of the day.

Another barrier to the adoption of these systems is their usability for both patients and professionals. The complexity of using these tools poses an obstacle for a portion of the patient population. The complexity of the data flow and doubts about consistent compliance with data protection standards are probably the main adoption barriers for professionals. It is particularly important that these tools and the data flow are as simple and clear as possible, aiming to be practically transparent for both the patient and the neurologist (16, 17). To overcome these barriers, it is necessary for the devices to have validation and usability studies in clinical practice with a sufficient number of patients and for practical recommendations and standardized protocols to be generated to optimize usefulness and ensure results for patients, caregivers, and physicians alike. Additionally, integrating these systems with hospital IT systems would be crucial to provide all stakeholders with a secure and transparent data flow.

This review focuses on the main commercialized devices for monitoring motor symptoms, which have been the most clinically validated and are recommended by the National Institute for Health and Care Excellence (NICE) (18). Additionally, all promising devices that could be useful in clinical practice and have received medical device certification, such as FDA or CE certification, have been considered and discussed in this manuscript.

## 2 Wearable systems recommended by NICE

In January 2024, NICE published an updated list of wearable devices that are conditionally recommended for use in clinical practice (18). The main aim of publishing this guideline was to emphasize the importance of tracking Parkinson’s disease symptoms to help clinicians make informed care decisions. However, this process is challenging in current medical practice due to the fluctuating nature of symptoms and the difficulty patients may have in accurately recalling or describing them. NICE identified a total of five technologies based on their validation and characteristics: PKG™, Kinesia 360™, Kinesia U™, PDMonitor™, and STAT-ON™. These are currently the only devices conditionally endorsed by NICE for this purpose. Table 1 shows a brief comparison between the devices described in this Section.

**Table 1:** Conditionally recommended devices by NICE for remote monitoring of Parkinson’s disease to inform treatment decisions.

Manufacturer	Device name	Device location	Detected symptoms	Algorithm	Number of patients trained for ML	Medical device certification	Cost according to NICE	Weakest points	Strongest points
PKG Health	PKG™	1 wrist sensor	ON/OFF, Bradykinesia (Upper limbs), Dyskinesia (Upper limbs), Tremor, Inactivity/Rest	Statistical method	–	FDA/CE Class IIa/TGA	225£ per report	Detection of only arm symptoms/Report	Usability
Great Lakes Neurotech	KINESIA360™	2 sensors (wrist/ankle)	Tremor, Dyskinesia, Bradykinesia, Mobility, Posture, Steps	Regression model	–	FDA/CE Class I/TGA	224£ per report	Usability	Gait data added to wrist information
Great Lakes Neurotech	KINESIAU™	1 wrist sensor	Tremor, Dyskinesia, Bradykinesia	Regression model	–	FDA/UKCA	64£ per patient	Detection of only arm symptoms	Usability
PD neurotechnology	PDMONITOR™	5 body-worn sensors	ON/OFF, Bradykinesia, Dyskinesia, Tremor, Freezing of Gait, Gait Parameters, Inactivity/Rest	ML in clinical and home environments	20 in clinical settings, 24 in home environments	CE Class IIa	350£ per month	Usability/Report	Comprehensive evaluation of symptoms
Sense4Care	STAT-ON™	1 waist sensor	ON/OFF, Bradykinesia, Dyskinesia, Freezing of Gait, Gait Parameters, Falls, Inactivity/Rest	ML in home environments	92 in home environments	CE Class IIa	1,600£ per year	Tremor not detected	Algorithm performance/single device

### 2.1 PKG™

The PKG™, from Global Kinetics Pty Ltd., is a wrist-worn device designed to continuously monitor the motor symptoms of Parkinson’s Disease. The patient uses the device for 6 days, and the data is then uploaded to a server where it is processed, and a report is sent to the neurologist. The device has been approved under CE (Class IIa), FDA (Class 2), and TGA.

The PKG™ algorithm was published in 2012 by Griffiths et al. (19). The authors introduced a method based on accelerometer signal analysis obtained from the wrist during 2 min windows. From this window, it is analyzed the frequency characteristics between 0.2 Hz and 4 Hz, the maximum acceleration reached, and the time without movement, from which two indices are generated. One is associated with bradykinesia (BKS) and the other with dyskinesia (DKS), which are then represented on a graph with interquartile ranges to determine the severity of one symptom or the other. There is no evidence of a training-evaluation data method, so it is not considered a machine-learning algorithm. Validation was performed through the median of all BKS samples over 9 h across 10 days and correlated with the UPDRS, obtaining a significant *r* value = 0.64, *p* < 0.0005. A third score called FDS was designed to measure motor fluctuations (20). This score, expressed as an algebraic combination of BKS and DKS, determines if a patient is fluctuating. The device has been widely tested in clinical conditions and compared with UPDRS (21) or diaries (8, 20). For example, the work of Santiago et al. (22) determined that in 41% of neurologists, PKG™ provided more information than classic routine visits. In a study by Nahab et al., the authors also indicate utility in clinical practice (23). Furthermore, the system has shown good results in usability (24), although there were some scores to be discussed. For example, only 27% of patients scored positively on the report of PKG, and 59% rated the use of PKG as valuable in offering useful information to clinicians. Interesting articles can be found, such as the one from Ossig et al. (8). In this study, the agreement was moderate to high in the total number of OFF and ON with and without dyskinesia (K = 0.404 in OFF with bradykinesia and K = 0.658 with ON with dyskinesia). However, a low to moderate agreement was found if the agreement on every single-hour-level (in OFF with bradykinesia K = 0.215 and in ON with dyskinesia correlation of K = 0.324). A similar situation is observed in Löhle et al. (25), where there is a moderate correlation between PKG and clinicians when total hours are compared (0.43 in bradykinesia and 0.51 in dyskinesia). However, a poor correlation is noted when outcomes are compared every 30 min (0.13 in bradykinesia and 0.21 in dyskinesia). According to Monje et al. (11), the PKG™ has been widely validated but needs further independent validation. In the same line, the NICE highlights in the published guideline that PKG is the device with more clinical validations but more evidence is needed.

### 2.2 Kinesia 360™ and Kinesia U™

Great Lakes Neurotechnologies (GLN) is the main manufacturer of Kinesia 360, a double-device system (wrist and ankle), and Kinesia U™, a wrist-worn device. The device incorporates a triaxial accelerometer and gyroscope. Among all the commercialized existing devices, GLN was the first company to achieve FDA 510(K) clearance as a tremor transducer device. In Europe, Kinesia 360 is considered a Class I device. Kinesia 360™ provides information about tremor, dyskinesia, slowness, mobility, posture, and steps (26). The quantification of bradykinesia conducted on an ankle-mounted device is based on the analysis of specific frequency characteristics derived from both the accelerometer and gyroscope and is calculated using linear regression models which are correlated with UPDRS scores (27, 28). The dyskinesia algorithm also relies on a linear regression model with sensors worn on the more affected side of the body (29). However, the number of features evaluated is significant, totaling 18. The obtained correlation with the modified Abnormal Involuntary Movement Scale (mAIMS) is significant (*r* = 0.77). Both models to determine bradykinesia and dyskinesia are more complex than the method presented by Griffiths et al. for the PKG due to the significant amount of features extracted from the accelerometer and gyroscope. The Kinesia 360 device also measures essential tremor, which was tested with 20 PD patients with intraclass correlation coefficients over 0.7 (30). Kinesia 360 has been evaluated in numerous studies and demonstrated its effectiveness in some therapies such as levodopa (31), rotigotine (32), deep brain stimulation (33), or subthalamic stimulation (34).

GLN also launched a device called Kinesia U™ for continuous monitoring but also for specific tasks. The main advantage of this new device was to eliminate the ankle-mounted device, improving the adherence of the patient to this technology by using only a wrist device. However, it is not clear how this affected the algorithm. The new app includes the capacity to fill in a diary and rate their symptoms. Results are provided in a 0 to 4 score indicating the severity of each symptom. In Pulliam et al. (31), it was shown that Kinesia presents a good accuracy for tremor, dyskinesia, and bradykinesia, being tremor and dyskinesia the best symptoms detected with the Area under curve (AUC) of 0.89 and 0.86, respectively. The bradykinesia algorithm has an AUC of 0.82 and a false positive rate of 0.34.

### 2.3 PDMonitor™

PDMonitor™ is a system composed of five devices designed to comprehensively characterize all motor symptoms of a Parkinson’s disease (PD) patient from any part of the body. The device, which is manufactured by PDNeurotechnology, is a CE Certificate device Class IIa. Each device includes an accelerometer, gyroscope, and magnetometer (7). This eliminates the need to choose the most affected side, allowing for the monitoring of movements from the upper limbs, lower limbs, and trunk. The system was designed within the PERFORM project (35, 36), and its algorithms are based on training a database of experts and utilizing machine learning algorithms. The algorithms are briefly described in different publications, including tremor (37), dyskinesia (38), bradykinesia (39), and Freezing of Gait (FoG) (40). Gait parameters and the ON and OFF states are also provided (41). Each algorithm employs a distinct classification method. For instance, the tremor detection algorithm uses Hidden Markov Models and achieves an accuracy of 0.87. The dyskinesia detection algorithm is based on a decision tree with a classification accuracy of 0.85. The bradykinesia detection algorithm utilizes Support Vector Machines and has a classification accuracy of 0.745. For this machine learning classifier, 20 patients participated in clinical settings and 24 in home environments. The algorithm for FoG, on the other hand, employs a Random Forest classifier achieving an accuracy of 0.96. The database was formed by 5 patients with FoG, 6 patients without FoG, and 5 healthy patients (40).

In another study in 2023, Antonini et al. published an article where the accuracy, sensitivity, and specificity of the different symptoms were evaluated against UPDRS and diaries. All the results on sensitivity, specificity, and accuracy for all the algorithms are over 0.8, except for the sensitivity of Gait (0.67). The accuracy and specificity achieve 0.96 or more in all the symptoms. Bradykinesia obtains a moderate-high correlation of 0.68 with UPDRS. In 2021, due to the appearance of COVID-19, a 2-cases study was presented showing the feasibility of PDMonitor™ for monitoring symptoms in home environments (42). PDMonitor™ was also evaluated against UPDRS, achieving moderate to high correlations in all their claimed detected movements/symptoms (41). This article questions the subjectivity of questionnaires, given that showing a device works does not need to correlate highly with questionnaires, which have doubtful outcomes. Although the device offers a comprehensive map of motor symptoms, there are doubts about the wearability of the device due to using five separate devices (13). However, in Antonini et al. (43), it should be mentioned that the scores obtained in wearability are significantly high except for two items: difficulty in putting on the device and that patients would wear the device if it was invisible. These items are of special importance because a patient in the morning usually suffers morning akinesia due to the deep OFF that they could suffer. Having to set up five sensors is an important barrier that could drastically reduce adherence to the technology and is something to consider.

### 2.4 Stat-ON™

STAT-ON™ is a medical device Class IIa which is worn on the waist (44). The device, which was designed under the project REMPARK (45, 46), has been commercialized by Sense4Care SL. The device aims to minimize the number of sensors while maximizing high accuracy by measuring symptoms from a position near the centre of the human body and using machine learning techniques trained with large databases.

The system detects bradykinesia (47, 48), dyskinesia (49), FoG (50, 51), gait parameters (48, 52), and falls (53), providing ON and OFF outcomes (54). The device is based on machine learning algorithms, more specifically in support vector machine classifiers and support vector regression models. The database was obtained in home environments, and the gold standard was the video and the UPDRS-III. For the validation of the ON and OFF algorithm, diaries were used, and a clinician called the patient every 2 h to ensure the motor state annotated by the patient, creating a robust database. A total of 92 PD patients participated in the database, and 10 extra PD patients participated in the dyskinesia algorithm. The algorithm for ON and OFF was evaluated with 91 PD patients in 3 studies (54–56) obtaining sensitivity and specificity values over 0.92. The bradykinesia algorithm was evaluated with 75 patients employing UPDRS subscales and achieving a moderate-high correlation of 0.67 *p* < 0.01 (57). The dyskinesia algorithm, evaluated with a leave-one-out method and 102 patients, achieved a sensitivity and specificity of 1 and 0.95 in trunk dyskinesia, 0.90 and 0.95 in strong dyskinesia in limbs and neck, 0.78 and 0.95 in mild dyskinesia in the trunk, and 0.39 and 0.95 in detecting mild dyskinesia in limbs [59]. Finally, the FoG algorithm was evaluated with 15 PD patients with FoG, achieving 0.92 and 0.87 in sensitivity and specificity, respectively.

In an Italian study performed by Zampogna et al. (58), the FoG and Dyskinesia were evaluated in 71 PD patients and an AUC was obtained of 0.92 and an accuracy of 0.8. The FoG evaluation obtained 0.83 on AUC and 0.81 on accuracy comparing FoG patients vs. PD patients without FoG. The number of FoG episodes AUC obtained a score of 0.87.

In Cabo-Lopez et al. (59), the correlation between STAT-ON™ and the clinical decision for considering a patient candidate for second-line therapy was 0.73 (*p* < 0.001) and a significant association in results between STAT-ON™ and the MANAGE-PD questionnaire (*p* = 0.004). Finally, a clinical trial with 84 PD patients comparing STAT-ON™ against diaries, having the UPDRS as the gold standard, obtained a correlation of 0.63 (*p* < 0.001) against the 0.24 obtained by the diaries (60).

## 3 Other promising devices for monitoring PD motor symptoms

In January 2023, the NICE recognized 5 technologies based on the information obtained from previous years. However, in the last 2 years, several technologies have emerged and obtained their medical device certification. It is fair to consider these technologies as valid as the previous ones, but considering that their validation in clinical studies is still far from the described technologies in the previous section. Table 2 provides a set of suggested guidelines that could help clinicians in selecting wearable sensors based on specific clinical needs in PD. These recommendations may assist in identifying the most suitable devices for different scenarios, considering factors such as symptom detection, usability, and patient characteristics.

**Table 2:** Guidelines for selecting wearable sensors in Parkinson’s disease.

	Wrist devices	Waist devices	Multiple sensor systems	Shoe sensors
Tremor	Yes	No	Yes	No
Bradykinesia	Indicative	Yes	Yes	Not validated
Dyskinesia	Indicative	Yes	Yes	No
Freezing of gait	No	Yes	Yes	Not validated
ON and OFF	Indicative	Yes	Yes	Not validated
Gait	No	Yes	Yes	Yes
Sleep disorders	Yes	Not comfortable	Not comfortable	No
Detection of early symptoms	No	Yes	Yes	No
Detection of candidates for second-line therapies	No	Yes	Yes	No
Psychiatric patient	Yes	No	No	Yes
Patient with advanced cognitive state	Yes	No	No	Yes
Rehabilitation	No	Yes	Yes	Yes
Patient with no capacity to walk	Yes	No	No	No
Usability	Very high	High	Very low	Low

### 3.1 Neptune™

Neptune™ from Orbit Health GmbH is a class IIa device that monitors bradykinesia, dyskinesia and ON/OFF states. The algorithm employed deep-learning techniques, specifically convolutional neural networks, for the detection of ON, OFF, and Dyskinesia states. The window size entered into the convolutional neural network is 1 min in length and obtained 0.654 accuracy in a three-class outcome problem. The sensitivity and specificity obtained for OFF was 0.64/0.89, for ON 0.67/0.67, and for Dyskinesia 0.64/0.89. However, the correlation between the complete data obtained and the MDS-UPDRS subscale 3.14 was high, with 0.83 for bradykinesia and with AIMS item 5 was 0.84 for dyskinesia detection. Correlations with lower windows (1 h, 30 min, 5 min, and 1 min) decrease but maintain a moderate/high correlation, going from 0.775 for dyskinesia and 0.735 for bradykinesia for 60 min to 0.703 for dyskinesia and 0.632 for bradykinesia with a window length of 1 min.

The main advantage of deep learning is the high performance of the classifier and the ability to compute complete raw inertial data without treating, extracting, and selecting the key features, being what is called a “black box” (61). However, this has been the focus of discussions due to the transparency of these methods in the field of computer science. Another characteristic of deep learning is that it is used to be a method for training large databases such as video or image with thousands or millions of signals (pixels) per sample. Unfortunately, a triaxial signal accelerometer in 30 PD patients is not considered a large database. This circumstance has the problem of overtraining a classification model leading to false positives in new evaluation data. Thus, more evidence is needed in this device to validate from a computer science point of view their approach.

### 3.2 PD-Watch™

PD-Watch from Biomedical Lab s.r.l. is a wrist-worn device classified as a Class I medical Device based on inertial sensors. The device is a 43 mm × 40 mm × 13 mm smartwatch and weighs 16 g. It has a battery life of 15 days. The data is recorded and then uploaded to a cloud server, from where it is processed and a report is returned. The device is capable of detecting ON/OFF, bradykinesia (62), tremor (63), dyskinesia (64), and inactivity with the absence of movement. One of the differences with other smartwatches devices is that PD-Watch can also detect dyskinesia severity. All the algorithms are based on extracting frequency features, and a structure of conditions that once met, the frequency response of the accelerometer is compared to a certain threshold (62). Results show more than 0.8 in sensitivity, specificity, and accuracy in all the algorithms. Further and external evidence is needed for this threshold-based algorithmic system.

### 3.3 Feetme™ and Nushu

Feetme™ from Feetme, is a wearable device that consists of two insoles, a device for connecting the insoles, and an app to manage the insoles. This device can continuously monitor movement and foot pressure in order to obtain gait parameters. The device includes a triaxial accelerometer and gyroscope and 18 capacitive pressure sensors (65). Feetme™ has received ClassIm in Europe and Class I FDA 510(k) exempt medical device.

The APP connects to the device which uploads the inertial and pressure data and processes it to provide different outcomes. Some of these outcomes are gait parameters such as stride velocity, cadence, stride length, step time, stride time, swing time, stance time, and single and double support time (66). All these features have been tested to evaluate stroke (67, 68), multiple sclerosis (69), or Parkinson’s Disease (66, 70). The device was evaluated against a recognized gold standard such as Gait-rite also in healthy adults (69, 71). Feetme™ has demonstrated to provide good results in tests such as the 6 min walk test (65). However, in the field of Parkinson’s Disease, Feetme™ has not shown any clinical utility for assessing motor symptoms such as bradykinesia, freezing, dyskinesia, or tremor.

Another similar intended-use device is Nushu, from Magnes, which has been registered in the FDA under a Class 2 device. The main difference is that Nushu are shoes instead of insoles and that the system uses a magnetometer but not pressure sensors (72). Values such as stride velocity, stride time, stride length, swing time, cadence, symmetry and variability of steps are provided. Nushu is based on inertial sensors (triaxial accelerometer and triaxial gyroscope) and a triaxial magnetometer. An orientation algorithm is set if it detects motion or not. The algorithm employed to estimate the orientation is based on Madgwick’s filter, which reduces computational burden compared to classical Kalman filters (73). A segmentation of the signal and event detection based on thresholds are executed to finally extract spatial and temporal gait parameters (74). Nushu’s algorithms also used SVM and reduced-SVM for detecting gait phases, which shows a high algorithmic level, which is used to detect Freezing of Gait and provide a vibrotactile biofeedback to avoid FoG on PD patients (75). The device has been only tested with healthy users, although authors claim that the device is under some clinical trials in the field of Parkinson’s Disease (72).

The device works with an app that allows configuring the type of activity to record. After the test or the recording, which is stored in an internal memory, the data is transferred via Bluetooth to the app and then uploaded to a cloud from where, through a dashboard, the results can be seen (76). Similarly to Feetme™, it is claimed the relation of gait parameters to motor symptoms of Parkinson’s Disease, however, further studies are needed to validate this idea rigorously.

### 3.4 Apple Watch based devices

In 2021, Powers et al. (77) published an article where a smartwatch named MM4PD could monitor tremor and dyskinesia in Parkinson’s Disease. The study was divided into three phases. The first one in clinics was video recorded with three movement disorders specialists rating. A subset of subjects participated in a 1-week out-of-clinic measurement period for obtaining data from daily living conditions. In the second phase, 225 PD patients participated, from which 143 were used to design the algorithm and 82 for validation. Finally, a third part was performed with 171 control users. For the gold standard, video was used, but also MDS-UPDRS-III was employed.

The device collected signals from a triaxial accelerometer and triaxial gyroscope at 100 Hz. The resting tremor algorithm was based on an analysis of the signal between 3 and 7 Hz in a 2.56 s window, but it’s not clear the classification method, which significantly detracts from the credibility of the method in the scientific field, as Bloem et al. reports (78). The algorithm classifies essential tremors into slight, mild, moderate, and severe tremors.

The dyskinesia algorithm uses a 10.24 s window overlapped every 2.56 s to not lose information between windows. The algorithm of dyskinesia is not explained although it is reported a low false positive rate despite a possible confusion with walking. Also, in 69 PD patients, the clinicians disagreed with the dyskinesia label.

A total of two solutions resulted from this method: StrivePD from Rune Labs and Parky from H2O Therapeutics. The two devices were FDA 510(K) cleared and claim tremor and dyskinesia in their intended use. Although the algorithms are the same and run on the Apple Watch, the layout of their solutions and their services are different.

On the other hand, NeuroRPM from NeuroRPM Inc. is another software that runs on an Apple Watch and besides detecting dyskinesia and tremor, it also detects Bradykinesia according to its intended use (FDA 510(k) Clearance number K221772). There is no scientific evidence apart from the summary report provided in the FDA clearance which shows a study with 36 PD patients and a sensitivity and specificity of 0.718 and 0.951 for tremor, 0.714 and 0.774 for bradykinesia, and 0.712 and 0.947 for dyskinesia. The outcomes are provided every 15 min and are binary for each of the three outcomes: tremor (yes/no), dyskinesia (yes/no), and bradykinesia (low to normal/severe). NeuroRPM has registered a clinical trial (NCT05680961) executed at the Parkinson’s & Movement Disorders Centre of Maryland. The lack of information on the algorithm is a major drawback, taking into account the significant amount of smartwatches that obtain these measurements.

### 3.5 Surface electromyography devices

Surface electromyography (sEMG) is a non-invasive technique used to measure the electrical activity produced by skeletal muscles, making it a valuable tool in the research and management of Parkinson’s disease (79–82). sEMG involves placing electrodes on the skin above specific muscles to detect the electrical signals generated during muscle contraction and relaxation. In the context of Parkinson’s disease, sEMG can be used to analyze the muscle activity patterns that are often disrupted by the condition (rigidity or dystonic dyskinesia).

sEMG can also help in monitoring tremors by providing detailed data on their frequency and intensity, or in assessing muscle rigidity and coordination (83). With the help of an accelerometer, the system could provide a comprehensive state of the patient (84). In the market, we can find Adamant Health (85), a company that commercializes a solution for capturing sEMG signals from different parts of the body with small wearable sEMG sensors and provide outcomes about the motor states of PD patients. Adamant Health has a wide scientific background in the recognition of motor symptoms in Parkinson’s Disease (81, 83, 84, 86–88). On the other hand, Paragit Neurotech provides a specific device with EMG that can be mounted on a patch at any part of the body (89). The device contains an accelerometer, a gyroscope, and an internal memory for data logging. The sensor fusion enables identifying the appearance of tremor, stiffness and according to the information on the webpage, bradykinesia and dyskinesia.

Other devices such as Ultium from Noraxon (90), Trigno from Delsys (91), Freeemg from BTS Bioengineering (92, 93), and Mini Wave from Cometa (94) have been used to characterize some motor symptoms or gait parameters in PD. These device systems are composed of several sensors and can be set at any part of the body. Given that there is no standardization on the point where the sensors must be located, these devices are mainly intended to be used in research and clinical studies. As far as authors know, only Adamant Health and Paragit Neurotech have incorporated algorithms that allow quantifying symptoms.

## 4 Other devices in the market for Parkinson’s disease

Wearable sensors have revolutionized the assessment and monitoring of gait, especially in the context of Parkinson’s Disease (PD). These devices offer a non-invasive, continuous, and objective means to capture detailed gait parameters in real-world settings, crucial for both clinical management and research. Companies like APDM Wearable Technologies, McRoberts, Gait Up, Moticon, Ephion Health, and MHealth Technologies have developed sophisticated wearable systems that are particularly useful in the management of PD.

APDM Wearable Technologies, now a part of Clario, offers a suite of wearable sensors named Opal V2C. The company’s flagship product, the Opal sensor, is a small, lightweight device that can be attached to different parts of the body to capture detailed movement data. A total of 24 sensors can be attached to different parts of the body. The battery life of each sensor is 4 days (8 h per day), and every sensor contains a triaxial accelerometer and gyroscope. The Opal V2C has been designed for specific tasks such as the Timed-up and Go test (95), 2 and 6 Minutes walking test, including open-ended walk and turns (96). Balance activities, sit-to-stand, 360-degree turns, and activity and sleep data in daily living activities are also included in the set of activities that can be recorded and analyzed. In the field of Parkinson’s Disease, Opal has been mainly used for the analysis of gait, being correlated with UPDRS subscales of gait or even correlating gait and balance problems with cognitive symptoms (96–98). However, although the device provides a lot of information from specific activities, balance, and gait parameters, there is no association with PD symptoms such as bradykinesia, freezing of gait, or tremor.

An interesting device to understand both the severity and distribution of dyskinesia in several parts of the body is LID-Monitor from ClearSky. This device is based on 6 sensors that are set on the head, upper and lower limbs, and chest (99, 100). The monitoring period is up to 24 h. The algorithm is based on an evolutionary algorithm and achieves results of AUC > 0.9 (99).

One of the most interesting companies that manufactures medical devices for gait is McRoberts B.V. This company has developed three types of sensors: Dynaport7, the MoveMonitor, and the MoveTest (101). The Dynaport7 is the latest device offering extensive communication options, including USB, Wi-Fi, and Bluetooth. It collects data and generates results in under 15 min. Measuring 106.6 × 58 × 11.5 mm and weighing 55 g, it features a triaxial accelerometer and gyroscope with a battery life of 5 days. Another device, the MoveMonitor, has a battery life of 14 days and is designed primarily for long-term monitoring of activity in home environments. Lastly, the MoveTest is intended for specific evaluations. It includes a package of six tests: the 6-Minute Walk Test, Sit-to-Stand Test, Gait Test (to assess gait quality and extract spatiotemporal gait parameters), Sway Test (to measure patient balance), the Timed Up & Go Test, and the Short Physical Performance Battery (a brief test assessing general movement including gait speed, chair stand, and balance tests).

As remarked, McRoberts’ devices are focused on the analysis of gait, postures, balance, and specific tests, but there is no association with motor symptoms of Parkinson’s Disease (102). However, the device is useful for understanding the progression of PD based on gait, balance, activity of the patient or sleep patterns.

Other interesting devices that have been used in the field of Parkinson’s are Physilog 6 from Gait Up and mTest3 from mHealth, which are inertial systems focused on assessment and rehabilitation. These devices are designed to provide detailed insights into the patient’s gait, posture, and overall mobility, but not motor symptoms.

In addition, OpenGo insoles from Moticon are notable for their emphasis on research. These smart insoles are embedded with sensors that measure pressure distribution and gait patterns in real-time. They are particularly useful in research settings where detailed biomechanical data is essential for understanding the progression of Parkinson’s disease and the impact of various interventions.

## 5 Discussion

In this paper, several sensors have been presented. Thus, when healthcare professionals have the chance or want to monitor a patient with Parkinson’s, many questions arise: which is the best one? Which sensor suits my necessities? Is it going to be reliable? How much time does it take to understand this new technology? Is the patient going to have good adherence to the monitoring tool? All these questions are difficult to answer because every study is completely different and there are different necessities. For example, if a healthcare professional wants to monitor ON and OFF fluctuations, the best sensors are those that offer a comprehensive view of different symptoms from different parts of the body. If the neurologist wants to measure tremors, then a wrist-worn device is the best option. Measuring, for example, dyskinesia, which happens in all parts of the body with only a shoe sensor or a wrist sensor, will lead to several false negatives. From the waist, it is feasible to detect many parts of the body due to the location close to the centre of mass, but some of the upper limb dyskinesia cannot be measured (103). Multiple sensor systems might provide a complete approach to choreic dyskinesia. On the other hand, wrist-worn devices can be an optimal solution for measuring tremor and getting an indicative estimation of the state of the patient. Wrist-worn devices have slightly better scores than waist devices in usability (104, 105), and this is an advantage for psychiatric patients or patients who do not want to use multiple or waist-worn sensors.

However, literature has demonstrated that waist devices outperform wrist-mounted devices in analyzing human movement in general. In Kluge et al. (106), sensitivity was 20% higher in waist-mounted devices, and in patients with diseases such as PD, this distance increased. The number of false positives due to random movements and that from the wrist it is not possible to measure specific movements from the body, increasing the false negatives, has been reported in several studies (107–111).

Another point is the number of sensors. The usability of a device is essential to keep good adherence of the patient and reduce the rejection rate in clinical studies. In Parkinson’s Disease and other neurodegenerative diseases, the use of numerous devices might be stigmatizing to the patient, and minimizing the number of sensors is crucial for social matters. However, although it is common to think that more devices are better for characterizing motor symptoms, which in some cases is true (112), the complexity of algorithms is also crucial. A good example is presented in Rodríguez-Martín et al. (113), where several classifiers and features of inertial signals are tested, and the performance of the algorithms increases based on the complexity of the algorithm.

Some studies compare some of the mentioned devices (51, 104, 105), but it is not clear against which gold standard they should be compared. As mentioned in this paper, questionnaires are subjective, and for example, UPDRS has demonstrated high inter-intrarater variability (11, 114), and diaries do not correlate enough with medical opinion (14, 15). Cabo et al. compared a wearable device against different scales concluding that current clinical scales are time-consuming and subjective compared to some objective wearable devices (59). Some studies with STAT-ON™, and that could be generalised to other devices, show that the amount of information obtained with wearables is higher with diaries given that patients do not recall to fill them in.

Another aspect is to remind that devices with an FDA or CE Certification only provide a guarantee of safety for the patient with very few clinical validation data presented to the authorities. In other words, a certificate does not guarantee that a sensor works accurately. A good example happens in the FDA medical devices classification, which only has a classification product for devices for monitoring Parkinson’s symptoms, which is the “tremor transducer” (GYD classification). However, although there is not a bradykinesia or dyskinesia classification, some devices such as PKG, Kinesia, or NeuroRPM have been accepted as devices to measure these symptoms.

There are three key challenges that wearable devices need to address. The first is accurately monitoring sleep patterns. Although accelerometers are commonly used to estimate sleep by detecting periods of minimal movement, they have significant limitations. These sensors cannot adequately distinguish between actual sleep and quiet wakefulness (e.g., watching a movie or being in an OFF state), as they only measure physical activity and do not capture the physiological changes that occur during different sleep phases. Zampogna et al. suggest using more advanced techniques, such as EEG, EMG, or electrooculography, to analyze REM and NREM phases; however, implementing these techniques in commercial devices remains a challenge (115). The PKG system, which also relies on accelerometers, has shown preliminary results that could be useful for sleep monitoring, although more evidence is needed to confirm its effectiveness (21, 116).

The second challenge, which still lacks sufficient evidence, involves the assessment of non-motor symptoms. While some reviews have highlighted the usefulness of certain wearable devices in different aspects, many current approaches rely on inertial systems to evaluate drowsiness or the amount of movement (117, 118). Additionally, some devices have started incorporating patient feedback to complement the data collected by sensors, which can provide extra information about symptoms such as stress or anxiety. Other methods, such as photoplethysmography, EEG, ECG, and electrodermal activity, have also been used to assess these conditions; however, further studies are needed to validate their effectiveness in this area (107).

The third challenge focuses on early detection and prediction of motor symptoms, which could greatly improve patient outcomes by enabling timely intervention. However, current advancements are limited to exploratory studies, and no commercial medical device with proven predictive capabilities exists yet (119, 120). Continued research and development are essential to make symptom prediction a clinical reality.

Wearable devices hold great potential for improving the monitoring of treatment in Parkinson’s Disease by providing continuous, objective data on patient response. Unlike traditional clinical assessments, which are limited to brief, periodic evaluations, wearables can track changes in real time, offering valuable insights into medication effectiveness and highlighting fluctuations that might be missed otherwise. This capability allows clinicians to make timely adjustments to treatment regimens, optimizing dosage and reducing the risk of adverse effects. Continuous monitoring can help identify specific periods of poor symptom control or medication wearing-off episodes, enabling more precise management. By integrating wearable data into clinical practice, we can move towards a more personalized and adaptive approach to treatment, ultimately improving patient outcomes and enhancing quality of life.

## 6 Conclusion

The aim of this paper is to provide comprehensive information and organize the numerous sensors available on the market. Devices for monitoring Parkinson’s Disease (PD) symptoms represent a significant advancement in managing the condition. These devices offer continuous, objective data on motor symptoms, which can help clinicians make better decisions and potentially improve patient outcomes. They provide detailed information on symptoms such as bradykinesia, freezing of gait, ON/OFF fluctuations, and dyskinesia over extended periods in real-world settings, enhancing diagnosis and monitoring. While classical methods have proven useful, they have certain limitations. The devices discussed in this paper can complement the information obtained from traditional clinical trials and studies.

However, several challenges need to be addressed for these devices to be widely adopted. More validation studies are necessary, and in many cases, external validation or validation by official authorities such as NICE, FDA, or EMA is required. Additionally, integrating these devices into hospital systems to ensure secure and transparent data flow remains a significant challenge.

Overcoming these barriers will optimize the usefulness of these tools and ensure better outcomes for patients, caregivers, and healthcare providers. This paper has presented several sensors, many of which have substantial scientific endorsement and are recommended by unbiased authorities such as NICE.
